# Efficacy and safety of polysaccharide iron complex capsules compared with iron sucrose in hemodialysis patients: study protocol for a randomized, open-label, positive control, multicenter trial (IHOPE)

**DOI:** 10.1186/s13063-021-05663-1

**Published:** 2021-10-10

**Authors:** Renhua Lu, Xu Zhang, Xudong Cai, Xiaoxia Wang, Hua Li, Li Wang, Yijun Zhou, Jianxiao Shen, Qian Liu, Haifen Zhang, Zhaohui Ni

**Affiliations:** 1 Department of Nephrology, Renji Hospital, School of Medicine, Shanghai Jiao Tong University, 160 Pujian Road, Shanghai, China; 2 Department of Nephrology, Taixing People’s Hospital, Taizhou, Jiangsu China; 3 Department of Nephrology, Ningbo Hospital of Traditional Chinese Medicine, Ningbo, Zhejiang, China; 4 Department of Nephrology, Tongren Hospital, Shanghai Jiaotong University School of Medicine, Shanghai, China; 5 Department of Nephrology, Sir Run Shaw Hospital affiliated to Zhejiang University School of Medicine, Zhejiang, Hangzhou China; 6 Department of Nephrology, Shandong Qianfoshan Hospital, Jinan, Shandong China

**Keywords:** Kidney failure, Hemodialysis, Anemia, Oral iron, Multicenter, Efficacy and safety

## Abstract

**Background:**

Anemia is one of the main complications of chronic kidney disease especially kidney failure, which includes treatment with erythropoiesis-stimulating agents and iron supplementation, including intravenous and oral iron. However, intravenous iron may pose limitations, such as potential infusion reactions. Oral iron is mainly composed of divalent iron, which can excessively stimulate the gastrointestinal tract. Iron polysaccharide complex capsules are a novel oral iron trivalent supplement with higher iron content and lower gastrointestinal irritation. However, since high-quality evidence-based medicinal support is lacking, it is necessary to conduct clinical studies to further evaluate the effectiveness and safety of oral iron polysaccharide complex in chronic kidney disease patients.

**Methods:**

This randomized controlled trial uses an open-label, parallel group design, where the efficacy and safety of maintenance hemodialysis (MHD) participants is evaluated. The experimental group is assigned erythropoietins and iron polysaccharide complex (two capsules each time, bid), and the control group is assigned erythropoietin and sucrose iron (100mg, 2w) injection. Participants (aged 18–75 years) undergoing maintenance hemodialysis were considered for screening. Inclusion criteria included hemoglobin (Hb) ≥110g/L and < 130g/L, transferrin saturation (TSAT) > 20% and < 50%, and serum ferritin (SF) > 200μg/L and < 500μg/L. Exclusion criteria included acute or chronic bleeding, serum albumin < 35g/L, hypersensitive C-reactive protein (HsCRP) > 10 mg/L, and severe secondary hyperparathyroidism (iPTH ≥ 800 pg/mL). Full inclusion and exclusion criteria are described in the “Methods” section. The primary endpoint is TSAT of the participants at week 12. Secondary endpoints include Hb, SF, hematocrit (Hct), HsCRP, pharmacoeconomic evaluation, drug costs, quality of life, and indicators of oxidative stress. The treatment will last for 24 weeks with a follow-up visit at baseline (within 7 days prior to initial treatment) and weeks 4, 8, 12, 16, 20, and 24 after initial treatment. This clinical research includes 9 hemodialysis centers in mainland China and plans to enroll 186 participants.

**Discussion:**

It is expected that it will provide strong evidence to reveal the clinical efficacy and safety of oral iron in the treatment of chronic CKD-related anemia in MHD patients through this clinical trial.

**Trial registration:**

Chinese Clinical Trial Registry ChiCTR2000031166. Registered on March 23, 2020

## Background

Kidney disease is a serious threat to the health of people globally. Seventy-nine percent of patients in China Dialysis Outcomes and Practice Patterns Study (DOPPS) had Hb < 12 g/dL, compared with 93% in Japan and 70% in North America [1]. According to the latest epidemiological data published in *The Lancet* in 2019, the prevalence of chronic kidney disease (CKD) among Chinese adults is 10.8% [2]. Anemia is one of the major complications in patients with chronic kidney disease, especially those with kidney failure undergoing maintenance dialysis. The incidence of anemia was 51.5% in 2420 participants with CKD as seen in results from a Chinese cross-sectional epidemiological study, published in 2016 [3]. The study showed a high rate of iron deficiency anemia in Chinese patients with CKD, and the degree of iron deficiency was relatively serious [3]. According to a survey conducted for CKD, the incidence of iron deficiency anemia after dialysis was more than 60% [4]. However, iron deficiency anemia is associated with reduced quality of life and an increased risk of cardiovascular disease, which seriously affects the physical and mental health of patients [5]. Several evidence-based data show that iron deficiency anemia results in poor prognosis in patients with kidney failure, and it is associated with the high rates of hospitalization and mortality in patients with kidney failure [6]. There are nearly 800,000 dialysis participants in China, and standardized CKD-related anemia treatment is imperative to slow down exacerbation of complications in patients with CKD post-dialysis. The diagnosis and treatment of CKD-related anemia have shaped clinical practice guidelines [7].

Iron supplementation is an important management strategy for CKD-related anemia. The 2020 edition of Chinese Medical Doctor Association guidelines recommends that hemoglobin levels and iron metabolism status should be identified first and addressed with potential causes of iron deficiency, before starting iron therapy [7]. Whether or not they are treated with recombinant human erythropoietin (EPO), patients with absolute iron deficiency should be treated with iron supplements. Also, patients with functional iron deficiency should be treated with iron supplements after weighing the benefits and risks of treatment. Commonly used iron agents are intravenous iron and oral iron. However, iron overload still exists in clinical intravenous iron therapy. Iron overload further causes increased levels of oxygen free radicals and increased oxidative stress. In 2011, the Japanese Society for Dialysis first warned about the potential toxicity of intravenous iron maintenance therapy [8]. The issue of iron overload continues to be addressed in several guidelines around the world [9]. Moreover, oxidative stress caused by intravenous iron is also a concern for nephrologists. Oral iron reduces the risk of iron overload and has little effect on oxidative stress, but the first and second generations of oral iron have obvious gastrointestinal side effects. Polysaccharide iron complex capsule is the third generation of oral iron agent that has been widely used in clinical practice due to its advantages of little or no stimulation of the gastrointestinal tract, few side effects, stable coordination, good solubility, and high iron content.

Although the PIVOTAL trial [10] has demonstrated the safety of relatively high levels of ferritin and TSAT with IV iron, iron therapy is still a challenge in the clinical treatment of CKD-related anemia; hence, it is necessary to conduct clinical studies to explore the optimal treatment of CKD-related anemia [7–9]. This study is the first multicenter clinical study of iron polysaccharide complex in CKD participants with maintenance hemodialysis. It aims to evaluate the clinical efficacy and safety of oral iron and to provide additional drug options in the treatment of anemia in CKD participants. This study will offer treatment alternatives for people on maintenance dialysis, especially home therapies such as peritoneal dialysis or home hemodialysis, in whom IV iron is inconvenient.

This clinical trial drug iron polysaccharide complex, produced by SPH Qingdao Growful Pharmaceutical Co. Ltd, has been approved by the CFDA (national medicine permission number H20030033) and is a new oral iron supplement.

## Methods

### Objective

The objective of this study is to compare the efficacy of oral iron polysaccharide complex and intravenous ferric sucrose infusion in maintenance hemodialysis participants with corrected anemia and to evaluate the safety and cost-effectiveness ratio of the two treatment regimens.

### Trial design

**Fig. 1:**
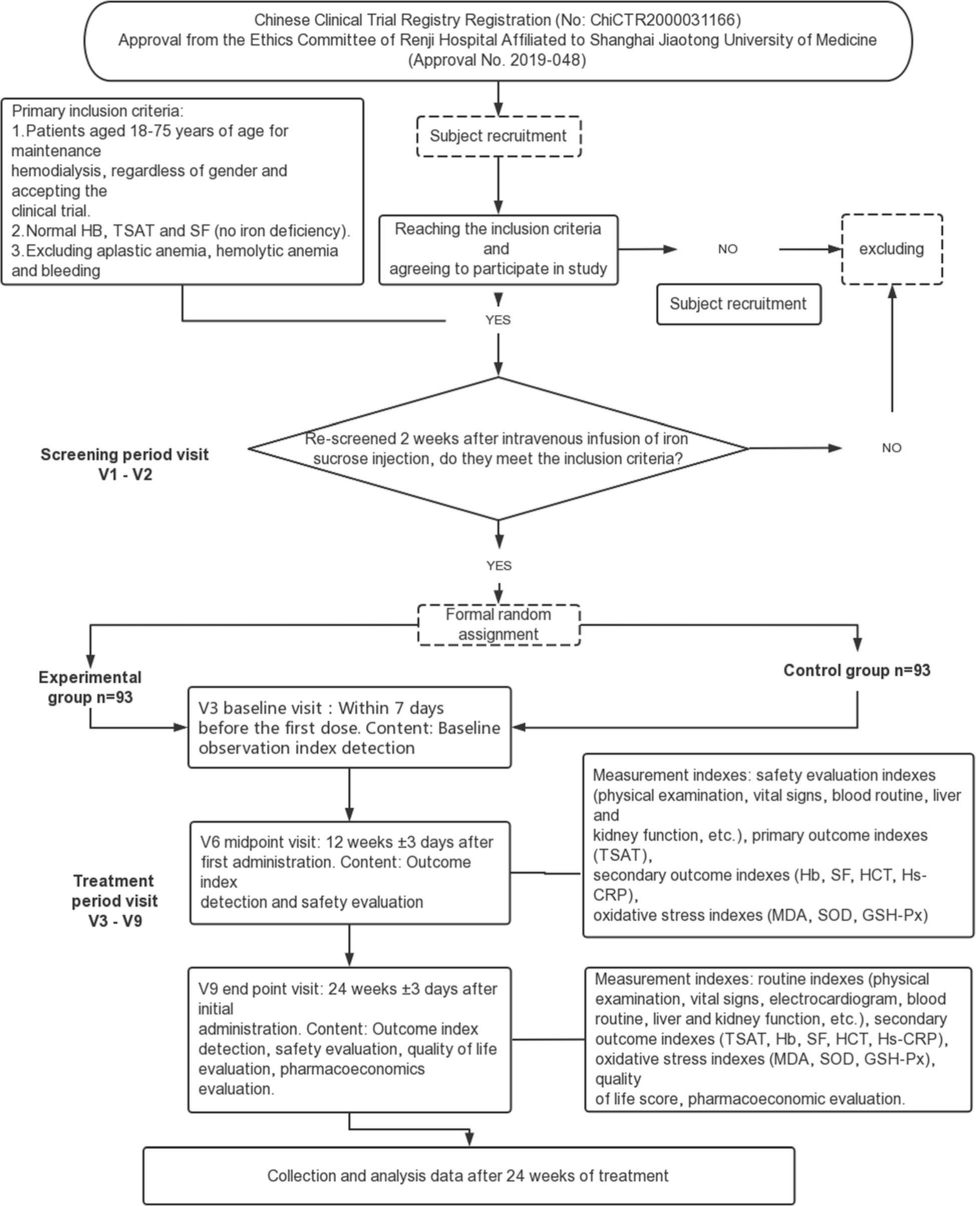
Flowchart of the test scheme. Experimental group [conventional drug (erythropoietin) therapy+ iron polysaccharide complex], control group [conventional drug (erythropoietin) therapy + iron sucrose injection]. Primary outcome: TSAT 12 weeks after treatment. Secondary outcomes: iron metabolism, oxidative stress, safety evaluation, pharmacoeconomic evaluation, quality of life score, etc.

This study is a multicenter, parallel controlled, randomized, open clinical trial (version 1.1; date 11 Dec. 2019, phase IV post-marketing) in China, which has been registered with the Chinese Clinical Trial Registry (http://www.chictr.org.cn/index.aspx) on March 23, 2020. The trial registration number is ChiCTR2000031166. The detailed flowchart is shown in Fig. 1. All eligible participants will be randomly divided into two groups in a 1:1 ratio: experimental group [conventional drug (erythropoietin) therapy+ iron polysaccharide complex] and control group [conventional drug (erythropoietin) therapy + iron sucrose injection]. Participants will complete the study visit at baseline (within 7 days before the first treatment) and weeks 4, 8, 12, 16, 20, and 24 after the first treatment. We used the SPIRIT reporting guidelines [11] in the current study.

### Sample size

This clinical trial set has a non-inferiority threshold of 7 (%) and a variance of 11 (%) for the positive control group. We predict a 2% real difference between the tested drug and positive control in transferrin saturation. The unilateral significance level is 2.5%, with an assurance of 80%. In the case of a 1:1 sample size between the control group and the experimental group, a total of 154 samples will be collected (77 of which will be in the control group and the experimental group, respectively). Considering a 20% drop-out rate, the actual planned inclusion number will be 186 cases.

### Setting and recruitment

**Fig. 2:**
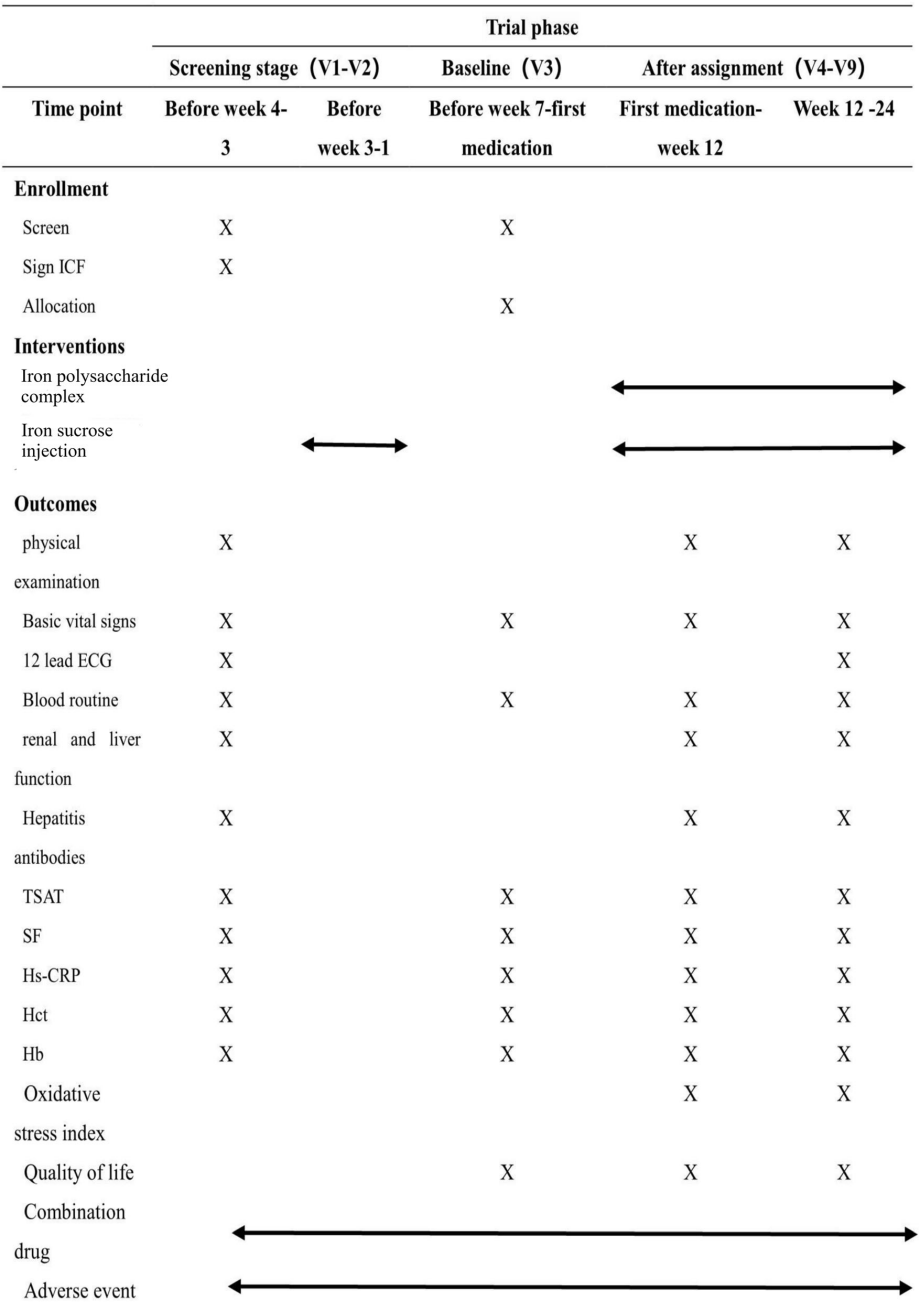
Schedule of enrollment, interventions, and assessments. ICF, informed consent form; ECG, electrocardiogram; TSAT, transferrin saturation; SF, serum ferritin; HsCRP, hypersensitive C-reactive protein; Hct, hematocrit; Hb, hemoglobin

A total of 186 subjects will participate in the study and will be recruited from 9 participating research centers (the hospital). Prior to recruitment, the investigator will carefully study the past condition, including but not limited to disease diagnosis and previous dialysis status of each participant. Researchers will fully consider the compliance of the participants within 24 weeks based on asking the participants. Informed consent forms (ICFs) will be obtained before collecting any participant data and participant information. After the participants sign the ICFs, the investigators will also sign the ICFs and record the informed consent process in the medical records. Hemodialysis participants eligible for inclusion in the trial will receive intravenous infusion (100mg total) of iron sucrose for 2 weeks after initial screening. If they still meet the inclusion criteria, they will be formally enrolled and started on medication. The treatment follow-up period of each participant is 24 weeks; the follow-up point of the screening and treatment period are shown in Fig. 2.

### Eligibility criteria

The target population in this study are participants undergoing maintenance hemodialysis and who can meet the following eligibility criteria.

#### Inclusion criteria

Participants must meet all the following criteria to be enrolled in the clinical trial [12]:

1. Age 18–75, either gender
2. Maintenance hemodialysis participants (≥3 months), dialysis frequency 3 times/week
3. Hemoglobin concentration ≥110g/L and <130g/L
4. Transferrin saturation >20% and <50%
5. Serum ferritin >200μg/L and <500μg/L
6. Used biosimilar epoetin and iron at least 12 weeks before enrollment
7. Signed ICF voluntarily

#### Exclusion criteria

Participants who meet any of the following criteria will not be eligible to participate in this clinical trial:

1. Iron allergy, allergic history, or intolerance to test drugs
2. Having erythrocyte aplastic anemia, other hematopoietic and hemolytic diseases
3. Acute or chronic bleeding
4. Serum albumin < 35g/L
5. Hypersensitive C-reactive protein >10 mg/L
6. Severe secondary hyperparathyroidism (iPTH ≥800 pg/mL)
7. Severe cardiac dysfunction (NYHA score > 3) and poor control of hypertension (systolic blood pressure ≥ 180 mmHg or diastolic blood pressure ≥ 100 mmHg)
8. Severe liver disease (ALT, AST, and TBIL ≥ 2 times the upper limit of normal) or hepatitis B surface antigen positive, or hepatitis C antibody positive
9. Malignant tumor
10. Severe bacterial or viral infection that occurred in the last 1 month, prior to signing the consent
11. History of blood transfusion in the last 1 month
12. Admitted to a hospital or planned a kidney transplant within the next 24 weeks
13. Pregnant or breastfeeding woman
14. Life expectancy less than 12 months
15. Participating or has participated in clinical trials of other drugs within the last 3 months
16. Gastric or duodenal ulcers or ulcerative colitis
17. History of chronic alcohol abuse, substance abuse. The presence of conditions that the investigator believes may affect participants’ compliance or test results

### Randomization and allocation

A central randomization method will be adopted in this study, and the principles of open and randomized control will be followed. All eligible participants will be randomly divided into an experimental group or the control group in a ratio of 1:1. An interactive web response system (IWRS) of the electronic data acquisition system (EDC) will be used to allocate and manage the random number, and the experimental drug (iron polysaccharide complex or iron sucrose injection) will be distributed according to the group corresponding to the random number. All information, including failure and success of participants screening, will be recorded, registered, and managed in the EDC system.

### Intervention

#### Experimental group

Iron polysaccharide complex (150mg) will be taken orally, 2 capsules per day, for 24 weeks. Participants will be treated with biosimilar epoetin (no need to dilute) intravenously with a single dose of 10,000U once a week during the maintenance period. At the same time, biosimilar epoetin dosage will be adjusted according to the measured hemoglobin concentration, as follows [7].

1. Discontinue biosimilar epoetin if Hb ≥ 130 g/L. Hb levels will be checked once every 2 weeks. If the Hb level drops to 110–130 g/L, the original biosimilar epoetin dose will be reduced by 25% or extended to once in 10 days for continuation.
2. If Hb < 110 g/L, TSAT, and SF are met, biosimilar epoetin will be administered as a single dose of 10,000U at least three times, every 2 weeks. Also, the Hb values of participants will be evaluated once every 2 weeks. The maximum dose of biosimilar epoetin will be 10,000U twice a week. If TSAT and SF fail to meet the standard, the iron deficiency will be corrected first, and the biosimilar epoetin treatment regimen will be adjusted once TSAT and SF are within the desired range.

#### Control group

One hundred milligrams of iron sucrose injection dissolved in 100 mL 0.9% sodium chloride solution will be intravenously administered at the end of dialysis once every 2 weeks for 24 weeks. During this period, participants will continue to receive intravenous biosimilar epoetin treatment in the same way as the experimental group. For the first intravenous infusion of iron sucrose injection, a small test dose will be performed. The test method refers to the drug instructions of iron sucrose injection, which are as follows.

1. Dosage: 1–2.5 mL (20–50 mg iron content) for adults. Children should be given 1 mL (iron content 20 mg) each time with body weight > 14 kg and given half of the daily dose (body weight kg × 1.5 mg/kg) each time with body weight < 14 kg
2. Caution: cardiopulmonary resuscitation equipment should be available during the test. If no adverse reactions occur after 15 min of administration, the remaining administration can be continued

### Dose adjustment within the next 24 weeks

If TSAT < 20% occurs during the test, the dosage of the iron agent will be adjusted as follows.

1. Iron polysaccharide complex: The iron polysaccharide complex prescription will be adjusted from one capsule daily to two capsules twice daily
2. Iron sucrose injection: the prescription of iron sucrose injection will be adjusted from “100 mg/time, intravenous drop, once every two weeks” to “100 mg/time, once a week.” If SF ≥ 500 μg/L and/or TSAT ≥ 50% after the above treatment, iron supplementation therapy will be stopped and SF and TSAT will be reevaluated twice a week. If SF < 200 μg/L and/or TSAT < 20%, or SF 200–500 mg/L and/or TSAT 20%–50% are found twice consecutively, iron supplementation therapy will be restarted. Iron supplementation therapy is iron sucrose injection 100 mg/time, intravenous drip, once a week

### Endpoints

#### Primary endpoint

1. TSAT will be measured at 12 weeks after baseline treatment.

#### Secondary endpoints

1. TSAT of participants’ changes between groups at 24 weeks of treatment
2. Hb, SF, Hct, and HsCRP values of participants’ changes between groups at 0, 12, and 24 weeks of treatment
3. Drug costs for participants’ changes between groups at 24 weeks of treatment (including cumulative biosimilar epoetin dose and iron supplementation)
4. Quality of life scale scores EQ5D-5L of participants’ changes between groups prior to treatment and at 24 weeks of treatment [12]
5. Indicators of oxidative stress, including malondialdehyde (MDA), superoxide dismutase (SOD), and glutathione peroxidase (GSH-Px), will be measured in participants’ changes between groups at 0, 12, and 24 weeks of treatment [12]
6. Cardiovascular events during the follow-up

### Withdrawal and drop-out

Participants can withdraw from the clinical trial at any time for any reason without prejudice to the investigator’s right to treat their disease. After fully considering the benefit of the participants, the investigator has the right to request the participants to withdraw for any reason, such as the occurrence of concomitant disease, adverse events [13], or treatment failure. The ethics committee reserves the right to request withdrawal of the participants for program violation, administrative reasons, or other valid and ethical reasons. All participants withdrawing from the clinical trial must complete a final trial evaluation. And the reason for withdrawal will be stated in the eCRF with all other appropriate and valuable information. Specific discontinuation and exit criteria are listed below.

Discontinuation criteria:

1. Participants not having used the drug even once
2. Without any test records or follow-up data, no safety and efficacy data records
3. Affecting the trial evaluation by using prohibited treatments and drugs in the protocol within the next 24 weeks
4. Other serious breaches of the scheme (participants with serious non-compliance to discharge criteria)

Exit criteria:

1. Exit requested by the participants
2. Recurring intolerable adverse reactions
3. SF ≤ 200 μg/L or TSAT ≤ 20% within 2 consecutive months
4. Hb concentration < 90 g/L or > 130g/L within 2 consecutive months
5. Poor compliance, failure to comply to protocol thus affecting efficacy
6. Study observations could not be continued due to unforeseen circumstances occurring during the test

### Statistical methods

The detailed contents of the statistical analysis methods of this clinical trial are as follows.

#### Data set category

1. Intention-to-treat (ITT): No one was excluded from the main analysis set after randomization
2. Per-protocol set (PPS): Good compliance case data that meets the main inclusion and exclusion criteria without affecting the main curative effects of prohibiting drugs within the next 24 weeks
3. Safety set (SS): Case data involving usage of the investigational drug product at least once, including safety evaluation

#### Statistical analysis technique

The number of participants selected and completing follow-up visits in the population and centers will be listed separately, and the three analysis data sets (ITT, PPS, SS) as specified above will be identified [14]. Cases of protocol violation should also be listed and the reasons indicated. In terms of outcome indicator analysis, continuous variable indicators will count the number of cases (*n*), mean ($\overline{x}$), standard deviation (*SMD*), median (*M*), minimum (*min*), and maximum (*max*). The difference between groups will be analyzed using Student’s *t*-test. Counting and grading data will be used to calculate the frequency and composition ratio. The *χ*^2^ test/chi-square test will be used for differences between groups. Unless otherwise stated, all statistical tests will be conducted in a bilateral manner. For example, if *P* ≤ 0.05, the difference between groups is statistically significant. Results of this clinical trial will be analyzed using SAS version 9.2 or above software.

### Harms

Intravenous iron has a rare anaphylactic reaction, so we will conduct a small dose test before the first administration to ensure the safety of the participants. Safety endpoints are related directly to changes in laboratory safety indicators and incidence of adverse events between the treatment and control groups during follow-up. These endpoints will be listed according to the treatment received and recorded in detail. Participants will be followed up in detail; if any complications arise, appropriate treatment will be provided in accordance with current routine medical procedures.

### Ethics and dissemination

This clinical study has been approved by the Ethics Committee of Renji Hospital, School of Medicine, Shanghai Jiao Tong University (approval number: 2019-048). Other participating sub-centers must also obtain ethics committee approval documents prior to the start of clinical trials. The Good Clinical Practice (GCP) principles and guidelines shall be strictly followed during the test implementation [15]. At the same time, any problems related to the clinical trial must be reported to the ethics committee in a timely manner, such as changes in the trial protocol or participants’ information page, serious adverse events, termination or early termination of the clinical trial, etc. After the publication of study results, the study report will be published in a peer-reviewed journal and/or at a national or international conference. All researchers involved in the design, writing, and discussion of this clinical trial protocol are listed as the authors.

## Discussion

Anemia is one of the complications in participants on maintenance hemodialysis. The deficiency of hematopoietic iron is an important reason for this complication. Therefore, iron supplements are important to correct CKD-related anemia [16]. The commonly used iron preparations in clinical practice include intravenous and oral iron agents. Compared to oral iron, intravenous iron has the advantages of faster onset and better absorption. But it also has some limitations, such as inconvenient use of intravenous iron, which requires relatively high time and place of administration. At the same time, the health economics evaluation found that intravenous iron is more expensive than oral iron [17]. Accordingly, the intravenous iron agent is inferior to the oral iron agent in terms of the economic and convenience aspect of the treatment. At present, the main oral iron agent is divalent iron agent, which has some shortcomings such as low iron absorption rate, large gastrointestinal stimulation response, and relatively slow onset effect, which affect the therapeutic effect to some extent [18]. Iron polysaccharide complex, as an oral iron trivalent supplement, has the advantages of high iron content (46%) and gastrointestinal metabolism in the form of iron molecules without containing free iron ions.

There is little domestic clinical evidence of iron polysaccharide complex in the treatment of CKD-related anemia, so the application value and advantages of iron polysaccharide complex in the treatment of anemia in maintenance hemodialysis patients cannot be fully confirmed [19, 20]. Therefore, it is necessary to carry out further clinical trials to clarify the efficacy and safety of iron polysaccharide complex in correcting anemia in the maintenance of hemodialysis participants. In this study, intravenous injection of iron agent—iron sucrose will be used as the control group, and it is assumed that the efficacy and safety of iron polysaccharide complex in anemia and in maintenance hemodialysis participants will be non-inferior to that of iron sucrose injection. The purpose of this study is to increase the head-to-head data of oral iron compared with intravenous iron treatment and provide high-quality theoretical support for the treatment of CKD participants with CKD-related anemia. It is expected that this clinical trial will provide strong evidence as to the safety level and efficacy of iron polysaccharide complex in the treatment of anemia in maintenance hemodialysis participants. This study will provide a stronger evidence base for the development of guidelines in the treatment of CKD-related anemia and the establishment of efficacious and safe alternative treatments for managing iron deficiency anemia in chronic hemodialysis patients.

## Trial status

The protocol version number is 1.1 and the date is 11 Dec. 2019. The recruitment date is 1 Apr. 2021, and the approximate date when recruitment will be completed is 31 Dec. 2021.

## Data Availability

The investigator must properly handle all the data obtained during the clinical trial, and truthfully record all adverse events and serious adverse events during the clinical trial, to ensure the rights and privacy of the participants participating in the clinical trial. In accordance with the regulations, the right to access all test records belongs to the National Medical Products Administration, hospital ethics committee, medical inspection authorities, project managers, clinical research associate, etc., who verify the accuracy of the original data and understand the progress of the test during the trial. If the original records cannot be effectively verified, the investigator must assist the inspector/auditor in further verifying the quality of the data. The group leader of this study has set up a data monitoring committee (DMC) for the purpose of ensuring the safety of participants and the quality of study data. The DMC is composed of clinicians and biostatisticians from the Clinical Center for Investigation, Renji Hospital, and School of Medicine, Shanghai Jiao Tong University, who are not involved in this study. After the publication of trial results, the trial report will be published in peer-reviewed journals and/or in national or international conferences.

## Abbreviations

MHD: Maintenance hemodialysis
Hb: Hemoglobin
TSAT: Transferrin saturation
SF: Serum ferritin
Hct: Hematocrit
HsCRP: Hypersensitive C-reactive protein
CKD: Chronic kidney disease
ICF: Informed consent form
EPO: Erythropoietin
IWRS: Interactive web response system
EDC: Electronic data acquisition system
MDA: Malondialdehyde
SOD: Superoxide dismutase
GSH-Px: Glutathione peroxidase
DMC: Data monitoring committee
ITT: Intention-to-treat
PPS: Per-protocol set
SS: Safety set
GCP: Good clinical practice
